# Novel collector design and optimized photo-fenton model for sustainable industry textile wastewater treatment

**DOI:** 10.1038/s41598-024-58610-w

**Published:** 2024-04-13

**Authors:** Heba A. El-Gawad, Montaser Y. Ghaly, N. F. El Hussieny, M. Abdel Kreem, Y. Reda

**Affiliations:** 1 Department of Engineering Mathematics and Physics, Higher Institute of Engineering, El-Shorouk Academy, Cairo, Egypt; 2 National Research Centre, Chemical Engineering and Pilot Plant Department, Canal High Institute of Engineering and Technology, Suez, Egypt; 3 Chemical Engineering Department, Canal High Institute of Engineering and Technology, Suez, Egypt; 4 Higher Technological Institute, 10th of Ramadan City, Egypt

**Keywords:** Wastewater treatment, Integrated unit, Classical processes, Developed oxidation processes, Solar photo-fenton process, Free hydroxyl radicals, Environmental sciences, Engineering

## Abstract

Textile industry wastewater containing toxic dyes and high COD poses environmental hazards and requires treatment before discharge. This study addresses the challenge of treating complex textile wastewater using a novel integrated system. The system combines sedimentation, screening, adsorption, and an optimized solar photo-Fenton process to provide a sustainable treatment solution. A novel parabolic collector with a larger absorber tube diameter enhances solar radiation utilization at lower catalyst concentrations. This design is versatile, treating all types of wastewaters, especially those that contain colors, smells, solid and suspended materials, in addition to its importance for the treatment of difficult substances that may be present in industrial and sewage wastewaters that are difficult to dispose of by traditional treatment methods. Multivariate experiments optimized key photo-Fenton parameters (pH, catalyst dose, etc.) achieving significant pollutant removal (85% COD, 82% TOC, complete color) under specific conditions (pH 3, 0.2 g/L Fe(II), 1 mL/L H_2_O_2_, 40 °C and 100 L/h flow rate after 60 min irradiation). Kinetic modeling revealed second-order reaction kinetics, and multivariate regression analysis led to the development of models predicting treatment efficiency based on process factors. The key scientific contributions are the integrated system design combining conventional and advanced oxidation technologies, novel collector configuration for efficient utilization of solar radiation, comprehensive process optimization through multivariate experiments, kinetic modeling and predictive modeling relating process factors to pollutant degradation. This provides an economical green solution for textile wastewater treatment and reuse along with useful design guidelines. The treatment methodology and modeling approach make valuable additions for sustainable management of textile industry wastewater.

## Introduction

Textile industry wastewater generated from dyeing and finishing processes poses a significant environmental challenge due to its high pollutant load and recalcitrant nature. It is characterized by high chemical oxygen demand (COD), intense coloration, and the presence of toxic synthetic dyes^1–3^. Conventional biological methods have limited efficiency for treatment of such wastewater. Although physico-chemical techniques like coagulation, precipitation and adsorption can remove some pollutants, they have disadvantages like sludge generation requiring additional treatment and disposal^4,5^.

Advanced oxidation processes (AOPs) using hydroxyl radicals have emerged as a promising technology for treating textile wastewater through oxidative degradation of organics. Processes such as O_3_/H_2_O_2_, UV/H_2_O_2_, UV/O_3_ and photocatalysis using TiO_2_ have been studied^6,7^. Among AOPs, the solar photo-Fenton process has attracted attention as a green, economical alternative leveraging natural sunlight and catalyzed H_2_O_2_ for wastewater treatment^8–12^.

Advanced oxidation processes (AOPs) offer promising wastewater treatment due to their high efficiency, broad applicability, rapid degradation rates, and environmental friendliness compared to other methods^13^. Though, their initial and operational costs can be high. Integrating AOPs with physicochemical and/or biological techniques offers a cost-effective and sustainable solution for wastewater remediation and clean water production.

Despite adsorption boasts advantages in simplicity and cost-effectiveness^13–15^, its limitations include incomplete pollutant removal, particularly for complex wastewater, slow processing times, and the need for continuous adsorbent regeneration. Integrating adsorption with AOPs can overcome this limitation by combining adsorption's removal capacity with AOPs' degradation capabilities for effective pollutant removal^16^.

AOPs can regenerate spent adsorbents in adsorption by degrading pollutants^17^. This combined approach can even be designed for simultaneous operation and improved mass transfer, leading to synergistic pollutant degradation^16,18,19^.

Despite prior work, major knowledge gaps persist in implementing solar photo-Fenton for textile wastewater treatment:

Lack of studies on an integrated system combining conventional and AOP technologies for overall effectiveness and cost savings^20^.

Limited reactor designs and non-optimal operational conditions leading to inefficient utilization of solar irradiation^21,22^.

Deficiencies in process optimization to maximize pollutant degradation through key factors like pH, catalyst dose, H_2_O_2_ concentration and temperature^23,24^.

Absence of kinetic analysis and predictive modelling relating process params to treatment efficiency^25,26^. For that reason, the objectives of this study are:

1. To design an innovative integrated wastewater treatment system incorporating sedimentation, screening, adsorption, and optimized solar photo-Fenton oxidation.
2. To implement a novel parabolic collector reactor enabling effective radiation capture.
3. To optimize the operational params of solar photo-Fenton through multivariate experimental design.
4. To perform kinetic modelling and develop efficiency prediction models for the process through regression analysis.

The integrated system and modeling approach aim to provide an economical green solution for textile industry wastewater treatment and reuse. For giving the research more credibility and realism, a real wastewater from an important local industry from an industrial area in Egypt for textile industries was selected as a case study. This wastewater was used without any dilution or pretreatment.

### The key innovations and contributions of this manuscript are:

- Design of an integrated wastewater treatment system incorporating both conventional processes (sedimentation, screening, adsorption) and advanced oxidation processes (solar photo-Fenton) for efficient treatment of textile industry effluent.
- Optimization of operational params for the solar photo-Fenton process, including irradiation time, pH, Fe(II) dosage, H_2_O_2_ concentration, temperature, and flow rate to achieve high removal efficiencies for COD, TOC and colour.
- Use of an innovative compound parabolic collector with increased absorber tube diameter for the solar photo-Fenton reactor. This allowed effective radiation absorption with lower catalyst concentrations, reducing capital costs.
- Kinetic analysis of COD removal indicating conformance to 2nd order reaction kinetics for the solar photo-Fenton process.
- Development of efficiency prediction models/correlations for the photo-Fenton process through multivariate regression analysis using least squares method. These models relate process factors like Fe(II) dose, H_2_O_2_, pH, temperature and flow rate to removal efficiencies.

The essence of the contribution lies in providing a comprehensive and optimized treatment methodology integrating conventional and advanced oxidation processes for effective treatment of recalcitrant textile wastewater. The integrated system design, process optimization, novel collector reactor configuration, kinetic modelling, and development of predictive models offer a valuable framework for industrial implementation of solar photo-Fenton for wastewater treatment and reuse in the textile sector.

## Experimental work

### Materials utilized

GP grad chemicals namely, ferrous sulfate (FeSO_4_・7H_2_O) which is named as Fenton reagent, sulfuric acid (H_2_SO_4_), (30% w/v) hydrogen peroxide (H_2_O_2_), activated charcoal (0.25 micro particle size), chlorine (Cl_2_) and sodium hydroxide (NaOH) were used without any purification were offered by Merck.

### Source of wastewater

This study utilized real wastewater obtained from an industrial textile plant in gathered from a plant in El Sadat City Desert, Menofia Governorate, Egypt as a case study. Approximately 1000 m^3^ of untreated wastewater per d was discharged into a nearby pond by the mill. The wastewater exhibited significant pollution levels, including high concentrations of suspended particles and organic contaminants. Samples were collected directly from the outlet pipe without any additional treatment. The characterization and analysis of these effluents, including params such as chemical oxygen demand (COD), total organic carbon (TOC), and other physicochemical properties, were conducted following the procedures described in Standard Methods for the Examination of Water and Wastewater (1998)^27^ and Standard Methods for the Examination of Water and Wastewater (APHA, AWWA, 2005)^28^. Table 1 exhibits some of the important properties and params of the wastewater.

**Table 1:** Assessing the characteristics of actual wastewater in relation to the limit values established in Egypt.

Permissible limits^29,30,31^	Param	Raw wastewater (Influent stream)	Treated effluent
1100	COD (mg/L)	2500	375
–	TOC (mg/L)	1570	282.6
600	BOD_5_ (mg/L)	225	98.34
–	BOD_5_/COD ratio	0.09	0.26
7	UV450 nm (1/m)	39	
5	UV552 nm (1/m)	44	
3	UV660 nm (1/m)	13	
6–9	pH	10	4
–	T. PO4^3–^, (mg/L)	23	2.613
100	TKN as N_2_, (mg/L)	260	3.64
100	Oil and grease***, (mg/L)	610	3.87
50	TSS, (mg/L)	600	4.2

### Set-up

**Figure 1:**
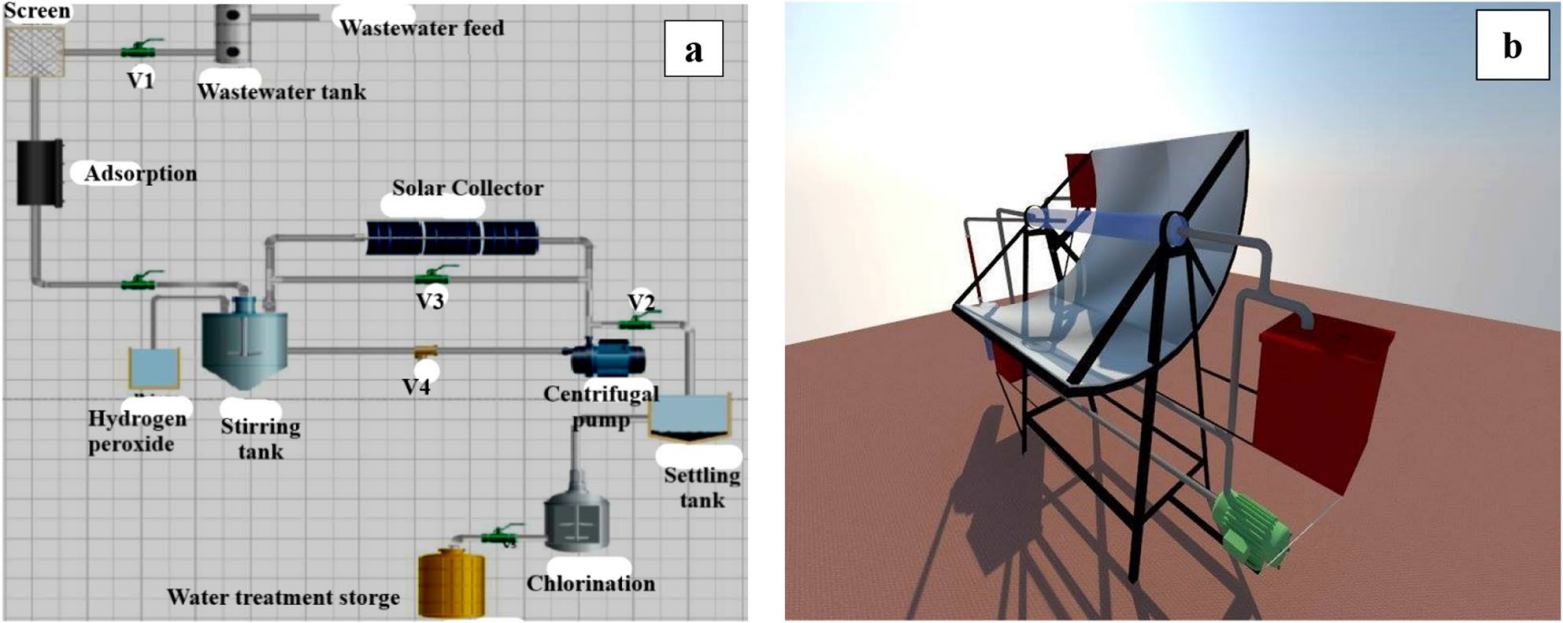
(**a**) and (**b**). Schematic diagram and a photo of the integrated designed experimental set-up.

In this investigation, a laboratory bench-scale unit was developed to treat wastewater effluent from a textile factory. The objective was to treat the wastewater and make it suitable for irrigation purposes in the large surrounding area. Figure 1a,b elucidates the schematic diagram of the process and a photograph of the integrated experimental setup that was designed for this purpose.

**Figure 2:**
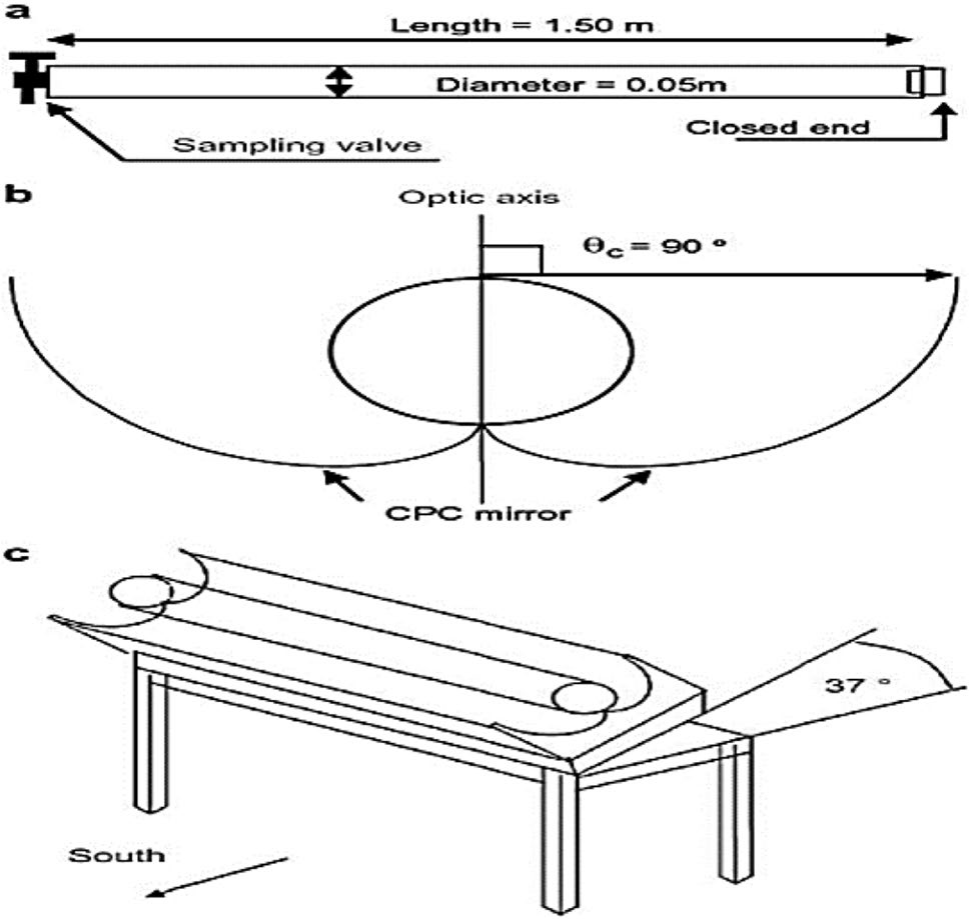
The components of concentrating parabolic collector (CPC): (**a**) Reactor tube, (**b**) CPC mirror, and (**c**) CPC reflector.

A bench-scale treatment unit designed for reusing treated textile wastewater for irrigation (Fig. 2) utilizes multiple stages. First, a 10 L propylene sedimentation tank removes large solids. A 3 cm diameter screener with fine pores (0.06–0.25 inches) captures floating pollutants. Activated carbon pretreatment (10 cm diameter, 26 cm height, 0.25-micron particles) removes color, odor, and organic load. The core treatment involves a solar photocatalytic oxidation unit that combines adsorption and oxidation of persistent pollutants. Wastewater is mixed with Fenton reagent (catalyst) and H_2_O_2_ (oxidant) in a 10 L mixing tank equipped with a mixer for homogenous blending. A non-concentrating parabolic reflector (4 m^2^ surface area) collects UV light. The reaction chamber is a 1.5 m long, 5 cm diameter transparent glass tube reactor tilted 30° south for optimal sun exposure^29,32^. A peristaltic pump circulates the wastewater through a closed loop around the illuminated area of the photoreactor. The flow system utilizes polytetrafluoroethylene or glass tubing and connectors.

### Procedures

#### Pretreatment steps

The real textile wastewater undergoes a multi-stage treatment process for progressive purification. First, sedimentation removes heavy particles, followed by screening to eliminate lighter matter. Activated charcoal filters then remove Odors and some organic pollutants through adsorption. Finally, the pretreated wastewater enters a storage tank where Fe(II) (catalyst) and H_2_O_2_ (oxidant) are added for the photo-Fenton process. Samples are collected throughout the treatment to monitor effectiveness (COD, TOC, colour).

#### Photo-catalytic oxidation step

For further analysis, a separate 7L batch of wastewater underwent photo-Fenton treatment in a laboratory unit. The wastewater's pH was adjusted and maintained using sulfuric acid or sodium hydroxide. The required Fenton reagent was mixed for 10 min in the illuminated section of the reactor. Hydrogen peroxide was continuously injected to sustain the reaction between H_2_O_2_ and Fe(II) ions. The initiation of the dosing pump and the start of hydrogen peroxide addition were considered as time zero. The dosage of hydrogen peroxide was calculated based on the stoichiometric ratio in relation to COD^30^. The solution was circulated at a flow rate of 100 L/h with a total suspension volume of 4 L in the entire system. The volume exposed to light (irradiated volume) was 3.5 L, which corresponds to the amount of solution held within the glass tubes. The experiment mimicked real-world conditions by utilizing natural sunlight between 11 AM and 3 PM during clear summer months (August–September). Solar radiation intensity was measured using a meteorological station located in the Solar Energy Department of the NRC in Egypt. Variations in sunlight intensity were accounted for by conducting and comparing results from multiple test sets. The UV intensity ranged between 3.5 and 4 W/m^2^, corresponding to roughly 30% of the total solar irradiation power.

### Analytical methods

To ensure accurate analysis and halt the ongoing photo-Fenton reaction, samples from each treatment step and the reaction vessel were collected regularly and immediately quenched with NaOH because solar photo-Fenton processes cannot occur at a pH above 10^33^. This step also neutralized any residual hydrogen peroxide, preventing further reactions with organic matter throughout the analysis process. TOC was measured using a Shimadzu-TOC analyzer 5000, pH with a WTW pH-meter 537, and COD using a Firma Lange LT 148 digester following standard protocols (details provided in the reference). Finally, color removal was determined by measuring absorbance at the dye's specific wavelength (λ_max_ = 530 nm) using a PD-303 UV spectrophotometer and calculating the concentration based on a calibration curve. This approach ensured accurate and well-controlled sample analysis throughout the experiment.

### Ethical approval

All authors have read this manuscript and would like to have it considered exclusively for publication in this journal. None of the material related to this manuscript has been published or is under consideration for publication elsewhere, including the internet.

### Consent to participate

All authors have read this manuscript and contributed to the study.

### Consent to publish

All authors would like to have it considered exclusively for publication in this journal.

## Results and discussion

### Pretreatment processes

Following a multi-step pretreatment (sedimentation, screening, and adsorption) as described in the experimental section, the studied wastewater achieved colour removal of 50%, COD removal of 25%, and TOC removal of 20% (Table 2). This treatment, while achieving some level of purification, falls short of the required removal efficiencies for safe disposal, necessitating further treatment with AOP. This finding highlights the limitations of standalone pretreatment in meeting stringent wastewater discharge regulations and emphasizes the need for additional, advanced treatment steps.

**Table 2:** Removal percentage of colour, COD, and TOC in numerous pretreatment processes.

process	Colour removal (%)	COD removal (%)	TOC removal (%)
Sedimentation	15	10	8
Screening	18	15	12
Adsorption	40	22	18

### Advanced oxidation process by solar photo-fenton process

The solar photo-Fenton process was selected as a homogeneous photo-catalytic advanced oxidation process for this study. The objective was to investigate the impact of several influential params on the efficiency of the process. These params include sunlight intensity, pH level, initial concentration of Fe(II), amount of H_2_O_2_, flow rate of the dye solution, temperature, and irradiation time. The aim was to determine the ideal operating conditions that would maximize the removal percentages of colour, COD, and TOC.

#### Effect of solar radiation

Prior to evaluation of solar decolorization and degradation of the wastewater using Dark Fenton process and photo-Fenton process, control experiment was carried out under sunlight in the absence of Fenton reagent. The experiment was conducted at the same operating conditions and same initial concentrations of COD, TOC, and pH. A control experiment under sunlight without Fenton reagent revealed minimal color reduction (less than 15%) and modest COD/TOC reductions (9% and 6%, respectively) after 180 min. This suggests the wastewater's resistance to solar photolysis alone. Interestingly, the presence of Fenton reagent in the dark (Dark Fenton) also led to lower removals of color, COD, and TOC. This might be attributed to the adsorption of pollutants onto the surface of Fe(II) ions in the Fenton process, which aligns with observations from previous research^34^. These findings emphasize the crucial role of the Fenton reaction in driving the significant pollutant degradation observed in the photo-Fenton process.

**Figure 3:**
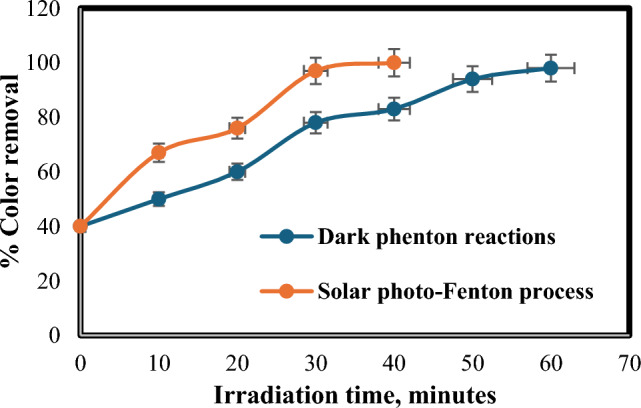
Influence of solar light on % colour removal: [Fe(II) = 0.2 gm/L, H_2_O_2_ = 1mL/L, temperature = 40 °C, and flow rate = 100 L/h].

**Figure 4:**
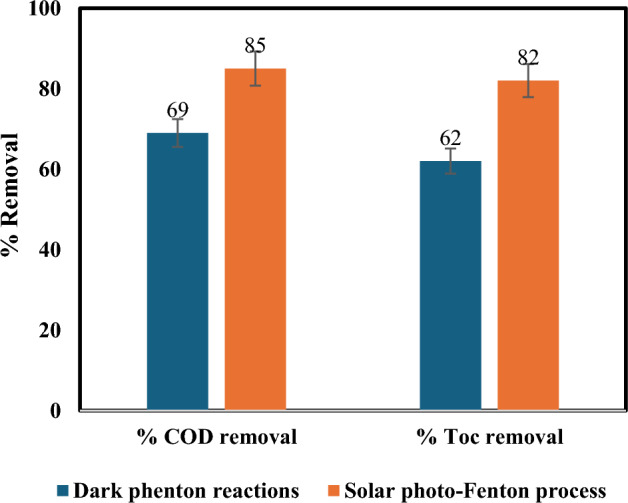
Influence of solar light on % removal of COD and TOC: [Fe(II) = 0.2 gm/L, H_2_O_2_ = 1mL/L, temperature = 40 °C, irradiation time = 60 min, and flow rate = 100 L/h].

Investigating the effect of solar light, two experimental runs were carried out using Fenton reaction one of them is in the dark and the other one in the presence of natural sunlight. Figure 3 elucidates comparison in the effectiveness for removal of colour against irradiation time with using the photo-Fenton process. Figure 4 depicts the effect of solar light in the photo- Fenton method on both COD and TOC removals comparing with that in dark process.

Balancing cost and efficiency are crucial in the photo-Fenton process. While shorter treatment durations are desirable for economic reasons, insufficient irradiation can leave behind partially degraded pollutants, hindering COD and TOC reduction. Additionally, high residual H_2_O_2_ at short treatment times can further decrease efficiency^35^. Conversely, extending exposure to UV light enables the generation of additional free radicals through various reactions, potentially enhancing degradation^36,37^. Our findings support this notion. As demonstrated in Fig. 3, complete color removal occurred within 40 min of solar irradiation compared to 98% removal after one h using the dark Fenton reaction. Similarly, Fig. 4 reveals that maximum COD and TOC removals (85% and 82%, respectively) were achieved at one h of solar irradiation, while the dark Fenton reaction attained 69% and 62% removal within the same timeframe.

#### Effect of pH

The photo-Fenton process relies heavily on pH for optimal hydroxyl radical formation, which are crucial oxidants. The acidic nature of the solution affects the rate of hydroxyl radical formation and the type of iron species present^38^. Acidic environments promote the generation of these radicals^39^.

**Figure 5:**
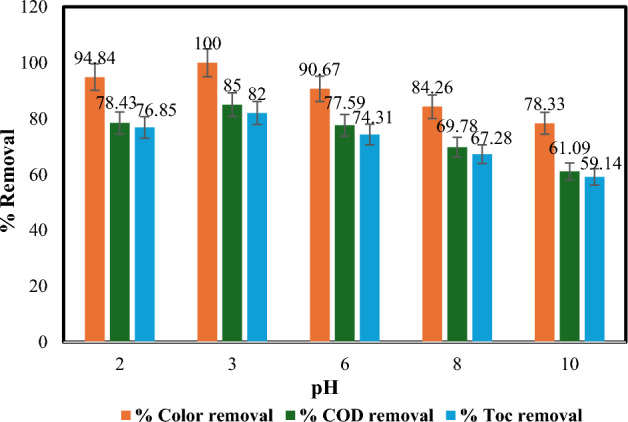
Influence of pH on % removal of colour, COD and TOC: [Fe(II) = 0.2 gm/L, temperature = 40 °C, irradiation time = 60 min, H_2_O_2_ = 1mL/L, and flow rate = 100 L/h].

To investigate the impact of pH, experiments were conducted at various pH levels (3–10) with fixed reaction time, H_2_O_2_ concentration, Fe(II) dose, temperature, and flow rate. As shown in Fig. 5, increasing pH from 3 to 10 leads to a decline in dye removal, COD removal, and TOC removal. This decrease is attributed to a combination of factors: reduced hydroxyl radical concentration at higher pH^36^, iron precipitation as Fe(OH)_3_ above pH 4 (limiting Fe(II) availability and light transmission), and the decomposition of H_2_O_2_ into water and oxygen by Fe(OH)_3_^40^. As well, hydroxyl radical oxidation potential weakens with increasing pH. Notably, dye removal efficiency drops significantly beyond pH 6 (reaching 67.45% at pH 10) due to the formation of ferric hydroxide complexes that hinder H_2_O_2_ hydrolysis by ferric iron. Our findings align with previous work by Lucas and Peres^41^ and Tabarek A. Sajjad^36^, who also identified pH 3 as optimal for dye removal. Lower pH may decrease activity due to the formation of various iron complexes in solution, affecting light absorption by Fe(II)^10,35^. Consequently, all experiments in this study were conducted at pH 3.

#### Effect of initial amount of Fe(II)

The solar photo-Fenton process, often employing sunlight or UV lamps as the photon source, relies on two crucial factors for optimal efficiency: hydrogen peroxide dosage and iron concentration. Hydrogen peroxide acts as an oxidant, vital for complete pollutant degradation. Conversely, iron facilitates the generation of highly reactive hydroxyl radicals (OH^•^) through the Fenton reaction, a process more efficient under acidic conditions^42–44^.

The solar photo-Fenton process relies on the generation of hydroxyl radicals (OH^•^) for efficient pollutant degradation. As depicted in Eq. (1), hydrogen peroxide reacts with Fe(II) salts to initiate this process. Further OH^•^ production can occur through the photolysis of various iron species and hydrogen peroxide (Eqs. 2–6)^12^. Maintaining optimal concentrations of both hydrogen peroxide and Fe(II) is crucial, as excess amounts can scavenge OH^•^ radicals, hindering overall treatment effectiveness.

1 $${\text{Fe}}^{{{2} + }} + {\text{H}}_{{2}} {\text{O}}_{{2 }} = {\text{Fe}}^{{{3} + }} + {\text{HO}}^{ \cdot } + {\text{HO}}^{ - } \left( {{\text{Acidic}}\;\;{\text{conditions}}} \right)$$

2 $${\text{Fe}}^{{{3} + }} + {\text{H}}_{{2}} {\text{O}} + {\text{ hv}} = {\text{Fe}}^{{{2} + }} + {\text{H}}^{ + } + {\text{HO}}^{ \cdot }$$

3 $${\text{Fe}}\left( {{\text{OH}}} \right)^{{{2} + }} + {\text{hv }} = {\text{Fe}}\left( {{\text{OH}}} \right)^{ + } + {\text{HO}}^{ \cdot }$$

4 $${\text{Fe}}\left( {{\text{OH}}} \right)^{{{2} + }} + {\text{hv}} = {\text{Fe}}^{{{2} + }} + {\text{ HO}}^{ \cdot }$$

5 $${\text{Fe}}\left( {{\text{RCO}}_{{2}} } \right)^{{{2} + }} + {\text{hv}} = {\text{Fe}}^{{{2} + }} + {\text{CO}}_{{2}} + {\text{R}}^{ \cdot }$$

6 $${\text{H}}_{{2}} {\text{O}}_{{2}} + {\text{hv}} = {\text{2 HO}}^{ \cdot }$$

Determining optimal reagent concentrations is often achieved through multivariate experimental design. This approach systematically varies hydrogen peroxide and iron concentrations while maintaining other params constant, allowing researchers to study their impact on degradation efficiency.

**Figure 6:**
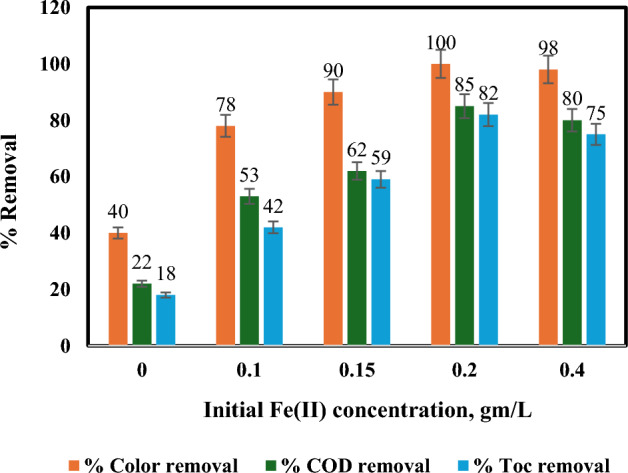
Influence of initial Fe(II) concentration on % removal of colour, COD and TOC: [pH = 3, temperature = 40 °C, irradiation time = 60 min, H_2_O_2_ = 1mL/L, and flow rate = 100 L/h].

Figure 6 exemplifies this approach, illustrating the influence of varying Fe(II) concentrations on COD and TOC removal, representing degradation and mineralization, respectively. Results showed that an Fe(II) concentration of 0.2 g/L achieved the highest percentage removals of COD (85%), TOC (82%), and colour (100%). Conversely, with no Fe(II) present, the removal efficiencies were significantly lower (22% COD, 18% TOC, and 40% colour) after 60 min using H_2_O_2_ under sunlight.

While higher Fe(II) concentrations generally enhance removal efficiency, an excessive presence can decrease mineralization yield. This decline is potentially attributed to the formation of short-lived intermediate iron(IV) species (Ferryl iron, FeO^2+^), which may hinder OH^•^ generation crucial for organic matter oxidation (Eqs. 7–9)^42^.

7 $${\text{H}}_{{2}} {\text{O}}_{{2}} + {\text{Fe}}\left( {{\text{II}}} \right) = {\text{FeO}}^{{{2} + }} + {\text{H}}_{{2}} {\text{O}}$$

8 $${\text{FeO}}^{{{2} + }} + {\text{Fe}}\left( {{\text{II}}} \right) + {\text{H}}^{ + } = {\text{Fe}}\left( {{\text{OH}}} \right)^{{{2} + }} + {\text{Fe}}\left( {{\text{III}}} \right)$$

9 $${\text{Fe}}\left( {{\text{OH}}} \right)^{{{2} + }} + {\text{H}}^{ + } = {\text{Fe}}\left( {{\text{III}}} \right) + {\text{H}}_{{2}} {\text{O}}$$

The observed increase in treatment efficacy with increasing Fe(II) concentration can be explained by the generation of more OH^•^ radicals through additional photo-Fenton reactions. This translates to a faster dye removal rate^45^. Secondly, higher Fe(II) doses not only ensure a complete redox reaction but also contribute to coagulation due to their presence, acting as both catalysts and coagulants^46,47^.

#### Effect of initial hydrogen peroxide concentration

**Figure 7:**
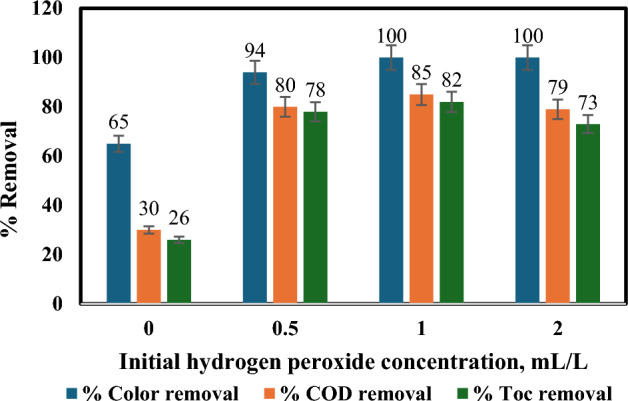
Influence of H_2_O_2_ amount on % removal of colour, COD and TOC: [pH = 3, temperature = 40 °C, irradiation time = 60 min, Fe(II) = 0.2 gm/L, and flow rate = 100 L/h].

To investigate the impact of hydrogen peroxide (H_2_O_2_) dosage on the photocatalytic degradation of wastewater using a solar photo-Fenton process, researchers conducted experiments with varying H_2_O_2_ concentrations while maintaining constant pH and initial Fe(II) levels. As shown in Fig. 7, increasing H_2_O_2_ from 0 ppm to 1 mL/L significantly enhanced the removal of COD, TOC, and colour due to increased generation of hydroxyl radicals^31,48,49^.

Increasing H_2_O_2_ dosage to 1.2 mL/L yielded minimal improvement, suggesting diminishing returns for the additional oxidant consumption. Notably, all treatments achieved near-complete colour removal (100%), significant COD reduction (85%), and acceptable TOC reduction (82%) within 60 min of irradiation. Interestingly, exceeding 1.2 mL/L H_2_O_2_ led to a slowdown in degradation, likely due to factors like H_2_O_2_ auto-decomposition into oxygen and water, OH^•^ radical recombination (Eqs. 10, 11)^24^ and scavenging by excess H_2_O_2_ itself^36,50,51^. Excess H_2_O_2_ reacts with OH^•^ radicals, competing with organic contaminants and reducing treatment effectiveness (Eq. 12)^24^. So, the optimal H_2_O_2_ concentration for this specific wastewater was determined to be 1 mL/L, with the caveat that the optimal dose can vary depending on the wastewater characteristics and iron concentration used.

10 $${\text{2H}}_{{2}} {\text{O}}_{{2}} = {\text{2H}}_{{2}} {\text{O}} + {\text{O}}_{{2}}$$

11 $${\text{2OH}}^{ \bullet } = {\text{H}}_{{2}} {\text{O}}_{{2}}$$

12 $${\text{OH}}^{ \bullet } + {\text{H}}_{{2}} {\text{O}}_{{2}} = {\text{HO}}^{ \bullet }_{{2}} + {\text{H}}_{{2}} {\text{O}}$$

#### Effect of temperature

**Figure 8:**
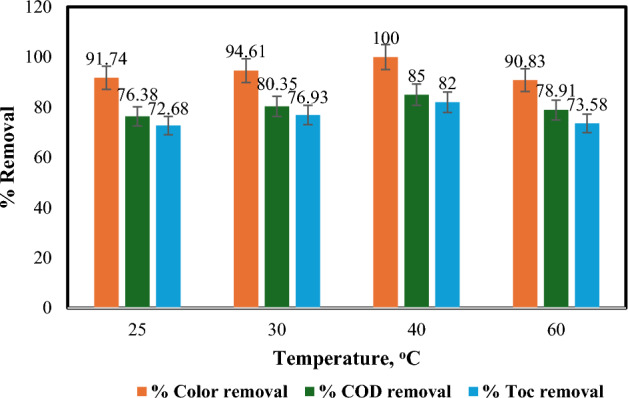
Influence of temperature on % removal of colour, COD and TOC: [pH = 3, H_2_O_2_ = 1mL/L, irradiation time = 60 min, Fe(II) = 0.2 gm/L, and flow rate = 100 L/h].

While the room temperature photo-Fenton process is common, investigating the impact of elevated temperatures relevant to the textile industry (60–90 °C) was crucial^41,52^. This study explored the effect of temperature (25–60 °C) on the removal of dye, COD, and TOC. Figure 8 reveals a significant rise in removal efficiency as the temperature increased from 25 to 40 °C, reaching a maximum of 100% for colour, 85% for COD, and 82% for TOC at 40 °C. This enhancement can be attributed to the accelerated interaction between organic material and hydroxyl radicals at higher temperatures^36^. Increasing the temperature to 60 °C resulted in a decrease in removal efficiency, likely due to hydrogen peroxide decomposition^53^, as depicted in Eq. (13). This emphasizes the importance of considering temperature's influence on both degradation and compound stability, even though higher temperatures can enhance reaction rates and generation of OH^•^ radicals^54^, these findings emphasize the importance of considering temperature's influence on both compound stability and degradation.

13 $${\text{H}}_{{2}} {\text{O}}_{{2}} = {\text{H}}_{{2}} {\text{O}} + 0.{\text{5O}}_{{2}}$$

Consistent with prior studies^36,55,56^, this investigation identified 40 °C as the optimal temperature for dye removal using the photo-Fenton process.

**Figure 9:**
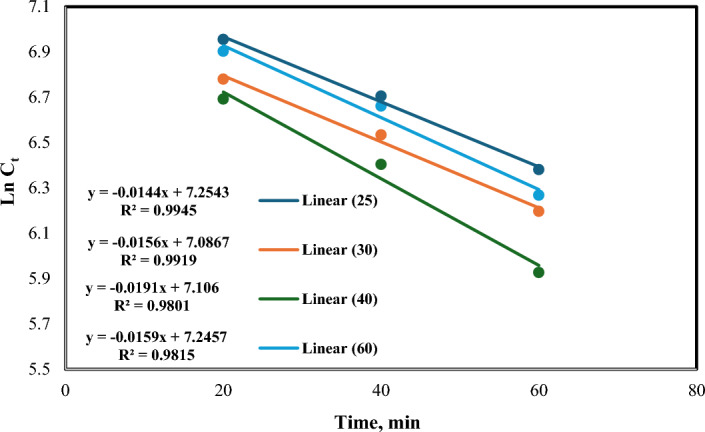
ln [C_t_] vs. irradiation time at different temperatures [pH = 3, H_2_O_2_ = 1mL/L, Fe(II) = 0.2 gm/L, and flow rate = 100 L/h].

Temperature also serves as a significant kinetic factor in boosting the colour and chemical oxygen demand (COD) removal from contaminated water. To assess the impact of temperature variations on the kinetics of COD removal, while maintaining other operational params constant, the treated solution was subjected to different temperatures. The findings depicted in Fig. 9 demonstrate an increase in the rate constant (k) as the reaction temperature rises from 25 to 40 °C.

**Figure 10:**
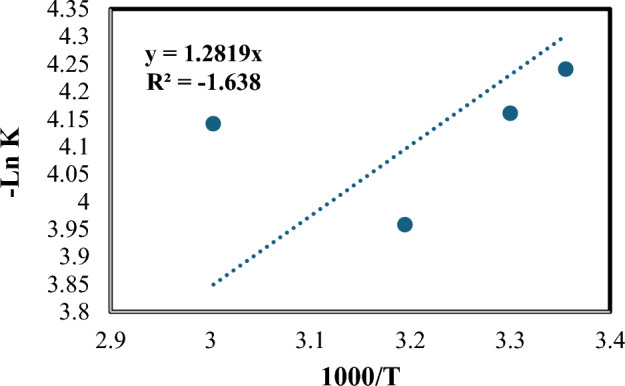
Arrhenius plot of COD removal [pH = 3, H_2_O_2_ = 1mL/L, Fe(II) = 0.2 gm/L, and flow rate = 100 L/h].

The relationship between the rate constant and temperature is described by the Arrhenius equation, which is used to model the effect of temperature on reaction rates. This relationship is illustrated in Fig. 10.

14 $${\text{k}} = {\text{A e}}^{{( - {\text{Ea}}/{\text{RT}})}}$$

where k is the rate constant, A is the frequency factor, E_a_ is an activation energy, and R is ideal gas constant. Activation energy calculated in plot was equal 10.66 kJ/mole.

#### Effect of flow rate

**Figure 11:**
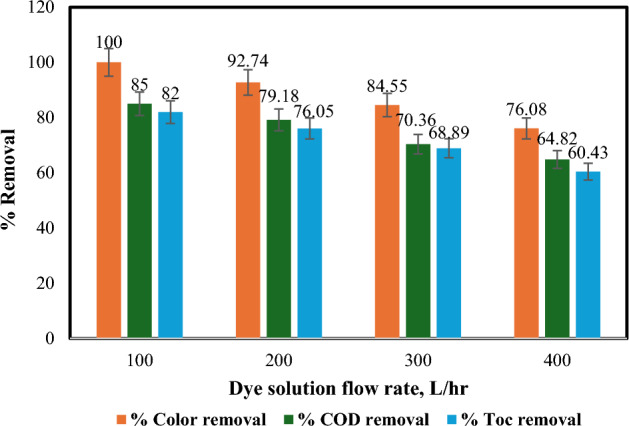
Influence of dye solution flow rate on % removal of colour, COD and TOC: [pH = 3, H_2_O_2_ = 1mL/L, irradiation time = 60 min, Fe(II) = 0.2 gm/L, and temperature = 40 °C].

To evaluate the impact of dye solution flow rate on dye removal, COD removal, and TOC removal percentages, the flow rate was varied between 100 and 400 L/h, which represents a pilot-scale process. The effect of flow rate on colour, COD, and TOC removal percentages was studied under the following conditions: pH 3, 40 °C, 60 min of irradiation time, 0.2 gm/L of Fe(II) dose, and 1 mL/L of H_2_O_2_ amount, as shown in Fig. 11. The highest colour (100%), COD (85%), and TOC (82%) removal percentages were achieved at a flow rate of 100 L/h. Figure 11 also demonstrated that increasing the flow rate from 100 to 400 L/h resulted in a decrease in colour, COD, and TOC removal percentages. This suggests an inverse proportionality between flow rate and colour removal efficiency. When the polluted solution's flow rate is increased, the UV light is exposed for a shorter period, necessitating a decrease in the amount of H_2_O_2_ to produce more hydroxyl radicals (OH^•^), as these results confirm^36,57^.

**Figure 12:**
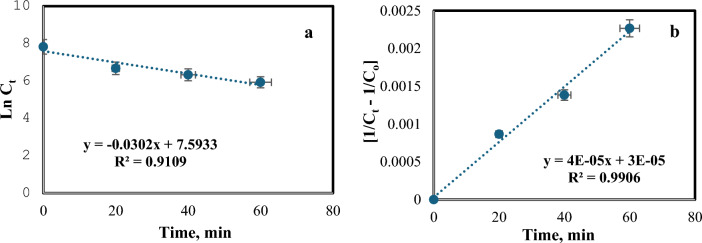
Reaction rate of 2500 ppm initial COD at ideal processing factors (**a**) 1st order kinetic model, (**b**) 2nd order kinetic model [pH = 3, H_2_O_2_ = 1mL/L, irradiation time = 60 min, Fe(II) = 0.2 gm/L, dye solution flow rate = 100 L/h , and temperature = 40 °C].

The data on COD concentrations were tested using various kinetic models to determine the best fit. The 1st and 2nd order kinetic models take on linear formulas that are expressed in the equations below:

15 $${\text{Ln C}}_{{\text{t}}} = {\text{K}}_{{1}} {\text{t}}$$

16 $$\frac{1}{Ct} - \frac{1}{Co} = {\text{K}}_{{2}} {\text{t}}$$

where C_o_ and C_t_ are the premier and final chromium concentrations, respectively. K_1_ and K_2_ are 1st and 2nd order rate constants in min^−1^ and L gm^−1^ min^−1^, respectively, and t is the treatment time (in min). Plots of ln C_t_ and [$\frac{1}{Ct}-\frac{1}{Co}$] versus time for each run resulted in straight lines, with slopes equal to the rate constants K_1_ and K_2_, respectively. Table 3 reveals adequate results for the COD removal kinetics. The results confirm that the COD removal kinetic obeys pseudo-2nd-order kinetics under ideal conditions (pH = 3, H_2_O_2_ = 1mL/L, irradiation time = 60 min, Fe(II) = 0.2 gm/L, dye solution flow rate = 100 L/h, and temperature = 40 °C), as the correlation coefficient R^2^ for the pseudo-2nd-order kinetic model fit the data better than the pseudo-1st-order kinetic model. Figure 12 elucidates the outcomes of the 1st and 2nd-order kinetic models.

**Table 3:** Reaction rate of 2500 ppm initial COD concentration.

Reaction rate equation	Kinetic equation	Rate constant	R^2^
1st order equation	ln C_t_ = 0.0302 t	0.0302 min^−1^	0.9109
2nd order equation	$\frac{1}{Ct}-\frac{1}{Co}=$ 4×10^–5^ t	4 × 10^–5^ L min^−1^ gm^−1^	0.9906

### Economical assessment

The economic evaluation of the project relies on estimating the capital costs associated with each replacement, considering the ongoing running and maintenance costs for different components. The projected profit was determined and evaluated in monetary terms to conduct a cost–benefit analysis.

For the treatment of 30 m^3^/d of wastewater, the estimated capital costs were $30,000. Additionally, the annual maintenance and operating costs for the complete integrated unit, including the solar photo-Fenton process, were calculated to be $9,000 per y.

## Statistic examination

The above results signify that the percentage removal of colour, COD and TOC is non-linearly influenced by various process factors, such as the Fe(II) concentration (mg/L), H_2_O_2_ amount (mL/L), solution pH, temperature, and flow rate (L/h). To elucidate the correlation between these process factors and the percentage removal of colour, COD and TOC, a statistical multivariate regression technique using the least squares method was applied. The mathematical models developed from this analysis are exhibited below:

17 $$\% {\text{ Colour}} = {27}.{895} + {41}0.{\text{95 A}} + {41}.0{\text{79 B}}{-}{2}.{\text{417 C}}{-}0.0{\text{33 D}}{-}0.0{\text{63 E}}{-}{675}.{\text{99 A}}^{{2}} {-}{12}.{\text{851 B}}^{{2}}$$

where A, B, C, D, and E are Fe(II) concentration (mg/L), H_2_O_2_ amount (mL/L), solution pH, temperature, and wastewater flow rate (L/h), respectively. The regression coefficient R^2^ of the resulting correlation was 94.7%.

**Figure 13:**
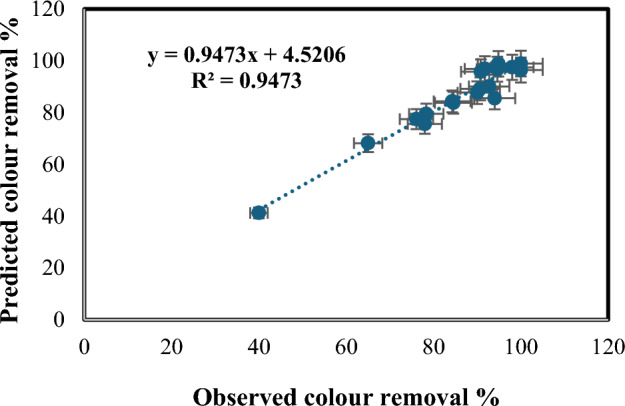
Experimentally determined colour removal % with the expected values.

**Figure 14:**
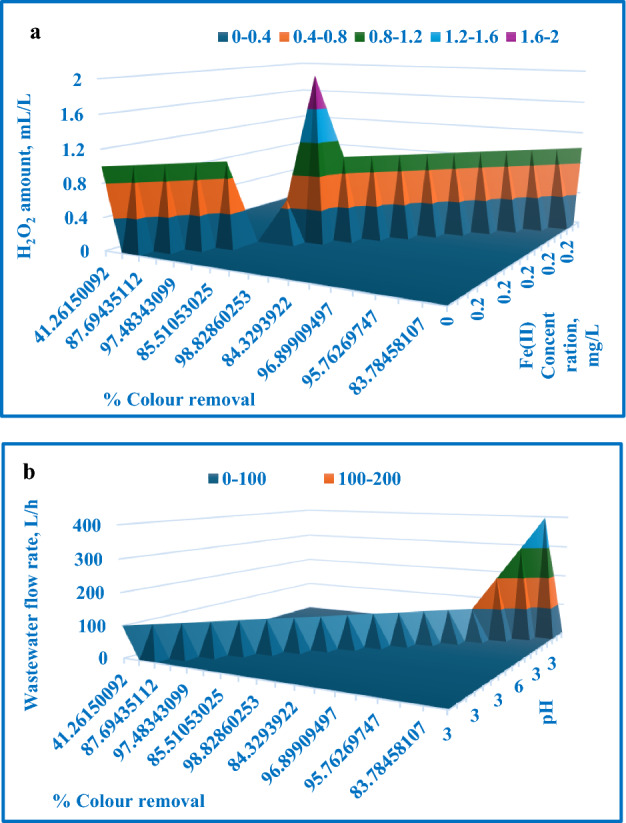
3D surface plot depicting the expected colour removal % in relation to: (**a**) H_2_O_2_ amount and Fe(II) concentration, (**b**) pH and wastewater flow rate.

A**** Table 4 displays the analysis of variance (ANOVA) data, while Table 5 contains the coefficient values, *p*-values, standard errors, and t-test results for each term. A *p*-value less than 0.05 reveals that the corresponding coefficient in the correlation is statistically significant. Figure 13 compares the experimentally determined colour removal percentages to the values predicted by the model. Figure 14 presents a 3D surface plot showing the expected colour removal percentage in relation to the various process factors. The first 3D surface plot (Fig. 14a) demonstrates the impact of Fe(II) concentration (mg/L) and H_2_O_2_ amount (mL/L) on the colour removal efficiency. The second 3D surface plot (Fig. 14b) showcases the influence of solution pH and wastewater flow rate (L/h) on the colour removal efficiency.

18 $$\begin{aligned} \% {\text{ COD }} = & \, - 15.295 \, + \, 457.58{\text{ A }} + \, 68.32{\text{ B }}{-} \, 2.315{\text{ C }} \\ & \quad + \, 0.043{\text{ D }}{-} \, 0.049{\text{ E }}{-} \, 754.542{\text{ A}}^{2} {-} \, 23.895{\text{ B}}^{2} \\ \end{aligned}$$

**Table 4:** ANOVA test outcomes for Fenton process [Colour removal].

	df	SS	MS	F	Significance F
Regression	7	3480.433	497.2047	25.67252	1.28E−05
Residual	10	193.672	19.3672		
Total	17	3674.105

**Table 5:** Values, *p*-values, standard error, and t-test for all coefficients [Colour removal].

	Coefficients	Standard Error	t-Stat	*P*-value	Significance
Intercept	27.89507298	9.062253	3.078161	0.011677	
X Variable 1	410.9508403	38.33564	10.71981	8.38E−07	Significance
X Variable 2	41.07940007	7.591638	5.411138	0.000297	Significance
X Variable 3	− 2.416535055	0.547241	− 4.41585	0.001303	Significance
X Variable 4	− 0.0324685	0.163803	− 0.19822	0.846847	Not significance
X Variable 5	− 0.063137432	0.013561	− 4.65578	0.0009	Significance
X Variable 6	− 675.9900379	88.11126	− 7.67201	1.7E−05	Significance
X Variable 7	− 12.85088375	3.510168	− 3.66105	0.004382	Significance

**Figure 15:**
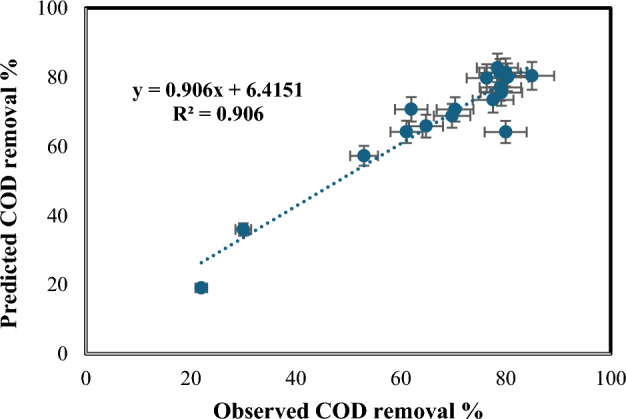
Experimentally determined COD removal % with the expected values.

**Figure 16:**
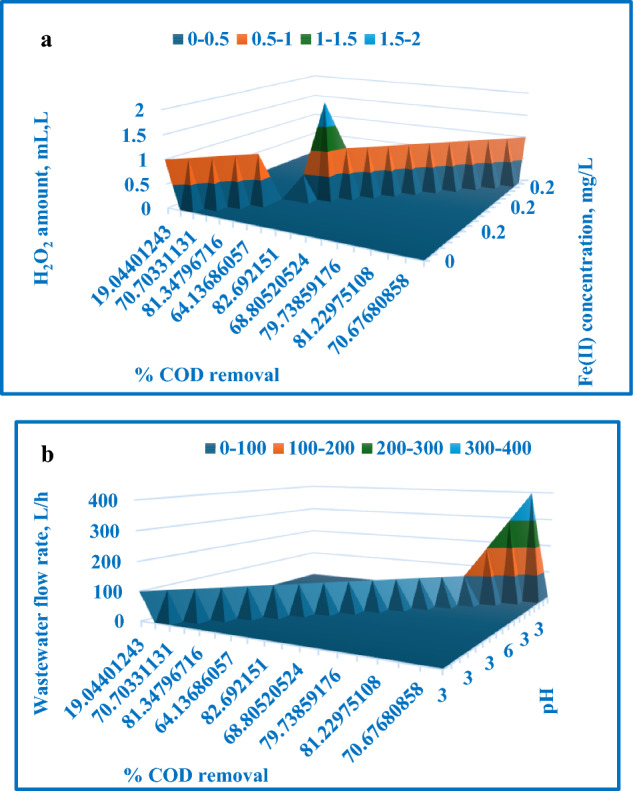
3D surface plot depicting the expected COD removal % in relation to: (**a**) H_2_O_2_ amount and Fe(II) concentration, (**b**)pH and wastewater flow rate.

The regression analysis resulted in a coefficient of determination (R^2^) of 90.6%, indicating a strong correlation between the process variables and COD removal percentage. Table 6 presents the analysis of variance (ANOVA) results, and Table 7 lists the coefficient values, *p*-values, standard errors, and t-test results. A *p*-value less than 0.05 represents a statistically significant term in the correlation. Figure 15 compares the experimentally determined COD removal percentages to the modeled values. Figure 16 exposes a 3D surface plot depicting the expected COD removal percentage as a function of the process factors. In Fig. 16a, the first 3D surface plot illustrates the relationship between Fe(II) concentration (mg/L) and H_2_O_2_ amount (mL/L), and their impact on the COD removal efficiency. Figure 16b, the second 3D surface plot, showcases the influence of solution pH and wastewater flow rate (L/h) on the COD removal efficiency.

19 $$\begin{aligned} \% {\text{ TOC}} = & - {21}.{53 } + { 88}.{\text{18 A}} + {183}.{\text{24 B}}{-}{2}.{\text{155 C}}{-}0.00{\text{4 D}} \\ & \quad {-}0.0{\text{48 E}} + {1928}.{\text{73 A}}^{{2}} {-}{184}.{\text{68 B}}^{{2}} {-}{4482}.{\text{88 A}}^{{3}} + {52}.{\text{4 B}}^{{3}} \\ \end{aligned}$$

**Table 6:** ANOVA test outcomes for Fenton process [COD removal].

	df	SS	MS	F	Significance F
Regression	7	4758.349	679.7641	13.76236	0.000211
Residual	10	493.9297	49.39297		
Total	17	5252.278

**Table 7:** Values, *p*-values, standard error, and t-test for all coefficients [COD removal].

	Coefficients	Standard error	t-Stat	*P*-value	Significance
Intercept	− 15.2952538	14.47223	− 1.05687	0.315434	
X Variable 1	457.5765893	61.22121	7.474152	2.13E−05	Significance
X Variable 2	68.32375037	12.12369	5.635559	0.000217	Significance
X Variable 3	− 2.31449096	0.873933	− 2.64836	0.024381	Significance
X Variable 4	0.042604552	0.26159	0.162868	0.873867	Not significance
X Variable 5	− 0.04850426	0.021657	− 2.23968	0.049031	Significance
X Variable 6	− 754.541756	140.7118	− 5.36232	0.000318	Significance
X Variable 7	− 23.8947676	5.605665	− 4.26261	0.001656	Significance

**Figure 17:**
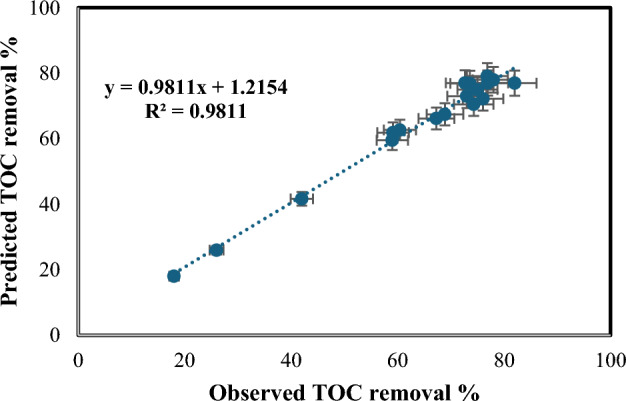
Experimentally determined TOC removal % with the expected values.

**Figure 18:**
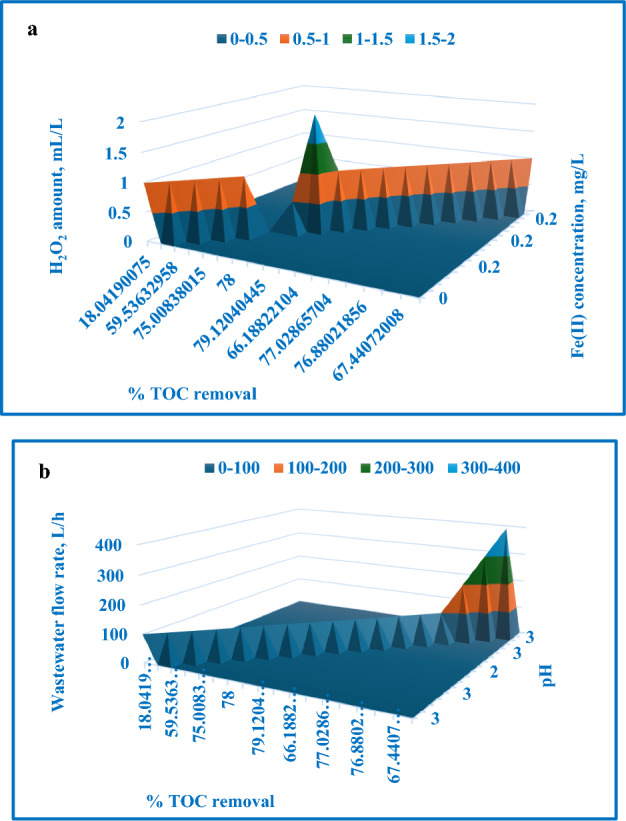
3D surface plot depicting the expected TOC removal % in relation to: (**a**) H_2_O_2_ amount and Fe(II) concentration, (**b**) pH and wastewater flow rate.

The regression analysis yielded a coefficient of determination (R^2^) of 98.1%, demonstrating a strong correlation between the process variables and the TOC removal percentage. Table 8 displays the analysis of variance (ANOVA) results, and Table 9 provides the coefficient values, *p*-values, standard errors, and t-test outcomes. A *p*-value less than 0.05 denotes a statistically significant term in the correlation. Figure 17 compares the experimentally determined TOC removal percentages to the modeled values. Figure 18 exhibits a 3D surface plot illustrating the expected TOC removal percentage as a function of the various process factors. The first 3D surface plot (Fig. 18a) demonstrates the impact of Fe(II) concentration (mg/L) and H_2_O_2_ amount (mL/L) on the TOC removal efficiency. The second 3D surface plot (Fig. 18b) showcases the influence of solution pH and wastewater flow rate (L/h) on the TOC removal efficiency.

**Table 8:** ANOVA test outcomes for Fenton process [TOC removal].

	df	SS	MS	F	Significance F
Regression	9	5504.245	611.5828	46.20846848	6.05E−06
Residual	8	105.8824	13.2353		
Total	17	5610.128

**Table 9:** Values, *p*-values, standard error, and t-test for all coefficients [TOC removal].

	Coefficients	Standard error	t-Stat	*P*-value	Significance
Intercept	− 21.5252439	7.629192	− 2.82143	0.022444346	
X Variable 1	88.18448855	104.6212	0.842893	0.02377083	Significance
X Variable 2	183.2365856	23.39839	7.831161	5.08989E− 05	Significance
X Variable 3	− 2.1553639	0.465499	− 4.63023	0.001687551	Significance
X Variable 4	− 0.0042411	0.135487	− 0.0313	0.975795114	Not significance
X Variable 5	− 0.0476216	0.01156	− 4.11952	0.003346415	Significance
X Variable 6	1928.73283	727.6999	2.650451	0.029235339	Significance
X Variable 7	− 184.674797	32.64527	− 5.65702	0.000477525	Significance
X Variable 8	− 4482.88389	1214.252	− 3.69189	0.006113556	Significance
X Variable 9	52.40325224	10.83225	4.837706	0.001291894	Significance

## Conclusion

This study presents a pioneering integrated wastewater treatment approach that addresses major gaps in implementing solar photo-Fenton oxidation for textile industry effluents. Unlike previous studies, it combines conventional pretreatment processes (sedimentation, screening, adsorption) with an optimized solar photo-Fenton advanced oxidation stage in a unified system. A key innovation is the use of a novel parabolic collector reactor with increased absorber tube diameter, enabling efficient solar radiation capture at lower catalyst doses—a substantial improvement over conventional designs.

Through comprehensive multivariate experimentation and optimization, remarkable treatment efficiencies were achieved—85% COD removal, 82% TOC removal, and complete decolorization under optimal conditions of pH 3, 0.2 g/L Fe(II), 1 mL/L H_2_O_2_, 40 °C, and 100 L/h flow rate after 60 min . Kinetic analysis revealed the pollutant degradation followed 2nd -order reaction kinetics. Moreover, this study derived efficiency prediction models through multivariate regression analysis, correlating process params like Fe(II) dose, H_2_O_2_ concentration, pH, temperature, and flow rate to the removal efficiencies. These models provide valuable guidelines for implementing solar photo-Fenton at an industrial scale for textile wastewater treatment.

The novel integrated system design combining conventional and advanced oxidation technologies, optimized photo-Fenton process, innovative reactor configuration, kinetic modelling, and predictive modelling frameworks constitute significant value additions. They provide a comprehensive green methodology for effective treatment and potential reuse of recalcitrant textile wastewaters. In contrast to conventional approaches limited by sludge generation and partial pollutant removal, this study offers an economical solution enabling sustainable industrial wastewater management.

With its multi-pronged treatment strategy, solar-driven process, reduced chemical usage through optimized operation, and valorisation of modelling insights, this work presents a transformative pathway for the textile sector. It paves the way for industrial implementation of solar-assisted advanced oxidation, ensuring regulatory compliance while promoting water conservation—a urgent need for developing economies with significant textile manufacturing. Overall, this study marks a notable advance in realizing the full potential of solar photo-Fenton for environmental remediation.

In summary, the key innovations are the integrated treatment system design, optimization of the solar photo-Fenton process, use of a novel collector reactor configuration, kinetic modelling, and development of prediction models—which provide an effective treatment methodology for textile industry wastewater. This approach offers an effective treatment methodology and valuable prediction models, contributing significantly to the field of industrial wastewater treatment.

## Data Availability

All the data and materials as well as software application or custom code support our published claims and comply with field standards.

## Abbreviations

COD: Chemical oxygen demand
BOD: Biological oxygen demand
TOC: Total organic carbon
TSS: Total suspended solid
TKN: Total Kjeldahl nitrogen
AOP: Advanced oxidation process
CPC: Concentrating parabolic collector
UV: Ultraviolet
